# Automatic brake Driver Assistance System based on deep learning and fuzzy logic

**DOI:** 10.1371/journal.pone.0308858

**Published:** 2024-12-31

**Authors:** A. R. García-Escalante, R. Q. Fuentes-Aguilar, A. Palma-Zubia, E. Morales-Vargas

**Affiliations:** 1 Tecnológico de Monterrey, Escuela de Ingenieria y Ciencias, Zapopan, Jalisco, México; 2 Tecnológico de Monterrey, Institute of Advanced Materials for Sustainable Manufacturing, Zapopan, Jalisco, México; Jinan University, CHINA

## Abstract

Advanced Driver Assistance Systems (ADAS) aim to automate transportation fully. A key part of this automation includes tasks such as traffic light detection and automatic braking. While indoor experiments are prevalent due to computational demands and safety concerns, there is a pressing need for research and development of new features to achieve complete automation, addressing real-world implementation challenges by testing them in outdoor environments. These systems seek to provide precise synchronization for decision-making processes and explore algorithms beyond emergency responses, enabling braking actions with short reaction times. Therefore, this work proposes a level 1 ADAS for automatic braking. The implementation uses an NVIDIA Jetson TX2 and a ZED stereo camera for traffic light detection, which, in addition to the depth map provided by the camera and a fuzzy inference system, make the decision to perform automatic braking based on the distance and current state of the traffic light. The contributions of this research work are the development and validation of a one-stage traffic light state detector using EfficientDet D0, a brake profile using fuzzy logic, and the validation with an on-road experiment in Mexico. The traffic light detection model obtained a mAP of 0.96 for distances less than 13 m and 0.89 for 15 m, with an average RMSE of 0.9 m and 0.05 m in the braking force applied, respectively. Integrated systems have a response time of 0.23 s, taking a step further in the state-of-the-art.

## 1 Introduction

The emergence of autonomous vehicles and other technologies such as the vehicular ad-hoc networks has effects in road safety and traffic efficiency [1, 2]. When talking about the levels of autonomy achieved, the research behind this becomes diverse and growing. Over time, the evolution of autonomous vehicles has forced the introduction of a classification and framework to categorize the levels of autonomy a vehicle can achieve. For this reason, the Society of Automotive Engineers, an organization dedicated to setting automotive standards, introduced a rating system in 2014. This system categorizes vehicles into six different levels from level 0 (No automation) to level 5 (Full automation), serving as a comprehensive guide to understanding the degree of automation within a vehicle [3]. Level 0 (No automation) places the entire responsibility for all driving tasks squarely on the driver. Level 1 (Driver assistance) provides certain features to assist the driver, but he remains primarily responsible for vehicle operation. Level 2 (Partial Automation) possesses a range of automated functions, yet the driver’s participation is still necessary for essential driving duties. In level 3 (Conditional Automation), a human driver does not need to maintain constant awareness of the vehicle’s environment. This is because the vehicle can independently manage specific tasks. Level 4 (High automation) indicates that the autonomous vehicle can assume control over all driving tasks but only within well-defined and specific scenarios. Finally, level 5 (Full Automation), here, autonomous vehicles can replicate all driving tasks in all possible scenarios.

The development of autonomous vehicles represents an important advancement in transportation technology; in this age, it is guided by industry leaders such as Google, Tesla, Audi, and Nvidia, involving powerful specialized processing hardware components such as Nvidia AGX Orin with 275 TOPS and 2048 Amperes cores for GPU, and 12 core A78 ARM V8 for CPU. The research commonly uses two critical subsystems: Perception and decision-making. The first interprets environmental and road data through cameras, proximity, and depth sensors. Then, the decision-making system processes the data to execute driving actions. In this context, research groups focus on improving traffic light detection, commonly implemented with single-stage detectors such as You Only Look Once (YOLO) and Single-Shot Detector (SSD) models [4–7]. On the other hand, the decision-making algorithms in the state-of-the-art commonly use strategies such as Deep Reinforcement Learning (DRL) and Fuzzy Logic (FL) to develop strategies for autonomous emergency braking systems and other driving maneuvers [8–11]. This research is commonly motivated by evidence associated with human error in driving, aiming to develop systems that reduce human errors and driving risks to enhance safety in driving and autonomous vehicles, prioritizing efficiency, safety, and mobility optimization.

However, most of the previous work was performed on desktop computers with high-performance graphics cards and in indoor environments, meaning there is no evidence that their traffic light state detector can work in a realistic environment where the resources are limited and without controlled factors. Therefore, we highlight the need for research and development of traffic light detection systems that are tested in outdoor environments, consider real-world implementation challenges, and provide accurate timing information for decision-making processes, additionally to the exploration of decision-making algorithms that go beyond emergency braking actions.

This work presents the development of a unified Advanced Driver Assistance Systems (ADAS) based on traffic lights using a Jetson TX2 and a ZED camera. The system works for those who drive on straight roads and can support multiple traffic lights. The results showed that it is possible to use Automatic Brake Driver Assistant System based on Traffic Lights (ABDAST) for velocities up to 10 km/h using a high-performance embedded system with reduced resources compared with those of higher monetary cost, such as the Jetson Orin. Key contributions include the development of a one-stage traffic light state detector using EfficientDet D0, the proposal of a Brake Profile Decision Making using fuzzy logic, and the validation of the model in on-road experiments.

## 2 Related work

In the realm of autonomous vehicles, the most advanced models documented in the literature are Google’s Level 4 autonomous vehicles [12], closely followed by Tesla’s Level 3 counterparts. These autonomous vehicles establish a blueprint that features a dual-subsystem architecture. The first component is the perception system, tasked with creating a detailed representation of the vehicle’s surroundings through sensor data [13]. The second component is the decision-making system, which uses data collected from numerous sensors within the vehicle to make informed decisions [14]. Following this, we provide a concise overview of the current state-of-the-art in autonomous vehicles, focusing on four key areas: traffic light detection and state classification, distance estimation, and braking systems. These are discussed in this section to understand the rationale of the decisions in this work.

State-of-the-art methods predominantly use two main approaches: model-based and learning-based methods, where learning-learning-based are the gold standard [15, 16]. e.g,. Ouyang et al. developed a real-time traffic light detector validated with on-road experiments. its approach uses a heuristic to identify the Region of interest (ROI) and locate the traffic light stages in an image with a small Convolutional Neural Network (CNN) for classifying traffic light stages [17]. Behrendt et al. [4] provided a three-step solution: the method starts by detecting the traffic light in the input image using YOLOV1. The second step involves classifying the traffic light state using a classification network, followed by tracking. In their work, Müller et al. introduced an SSD Inception V3 model (10 FPS, Nvidia Titan XP) for traffic light state recognition by incorporating a CNN with a modified loss function to improve the results [7]. On the other hand, Possatti et al. used a YOLOv3 to train a traffic light detector model with the IARA-TLD dataset, obtaining an mean Average Precision (mAP) of 55.21%. Then their approach used prior maps for traffic light state recognition [5] (16 Hz, Nvidia Titan XP). Weber et al. presented a singular CNN approach for detecting and classifying traffic lights at 33 hz in images of 640 × 480 pixels, obtaining a precision of 49.24% in their proprietary data set [18]. On the other hand, Lee et al. proposed a traffic light detection model that uses the ResNet-101 to create a single feature map [19]. Finally, the created feature map is used to detect traffic light stages. The proposed method achieved a mAP of 0.5681 and an Area Under the Curve (AUC) of 0.5973 in the Bosch Small Traffic Light Dataset (BSTLD) dataset with an Intersection Over Union (IoU) of 0.5.

Hirabatashi et al. [20] presented an approach to detect traffic lights and classify their state. First, an ROI with a traffic light is extracted using a camera, LIDAR, a localization method, and a map. Second, ROI is fed into a classifier to discriminate between the traffic light states. The authors obtained for distances up to 90 meters an AP of 97% and a recall of 90% in a proprietary dataset. Gao et al. [21] implemented a detector of traffic light stages. This solution comprises three stages: First, ROI extraction, followed by two detectors, a classical detector and a learning detector, to determine the light state. Finally, a scheduling algorithm is used to combine the two detectors, obtaining an accuracy of 79.6% at a speed of less than 25 km/h.

Wael et al. introduced a perception system that relies on a pre-trained YOLOv3 model to detect traffic lights [6]. They found that the ZED Camera used can estimate distances of less than 24 meters in outdoor conditions, while the method can effectively classify traffic light states at short distances. Then, Cabrera et al. presented a four-stage system. The first stage deals with the light variations using a PID controller. The second stage is color segmentation based on fuzzy logic clustering [11]. The third stage is the feature extraction of the traffic light stages, which uses morphological operations followed by filters. The fourth stage performs distance estimation using inverse perspective mapping, Kalman, and particle filters (10 Hz, Intel CPU). The system can run at 10 Hz with 99.4% accuracy in the range of 10 to 115 meters.

Decision-making algorithms for brake actuation such as [8] presented a decision-making strategy for vehicle autonomous emergency braking via deep reinforcement learning, or as presented on [9, 10] works, where each shows a trained model predictive control system strategy implemented on autonomous vehicles for braking action for collision evasion. For example, [22] proposes an algorithm for assessing threats in various traffic scenarios by determining the appropriate intervention type, whether it be steering or braking. However, Fuzzy logic has been applied previously in autonomous vehicles for decision-making in lane changing [23, 24], motion planning [25], and color space separation for traffic light states [11], even for collision avoidance decision of autonomous ground vehicles in controlled environments with an ethical approach [26].

Most of the motivation for both industry and academia has been working on solutions to develop ADAS that enrich vehicle perception, improve control decisions, implant vehicle intelligence, and refine communication technologies, all related to minimizing human errors that cause fatalities during conduction, reducing the cost of travel and improving the experience [27–30]. In recent years, annual global collision statistics have revealed more than 1.2 million reported deaths and 50 million incidents resulting in nonfatal injuries [31]. One of the causes of crash incidents is the driver inattention [32]. Additionally, the study of [33] associates crashes with slow Reaction Time (RT). This study found that fully attentive drivers typically exhibit RTs ranging from 0.70 to 0.075 seconds. For unexpected but regular scenarios, such as when the brake lights of a leading car are activated, the estimated RT is around 1.25 seconds, while for more surprising events, it extends to approximately 1.50 seconds. This is a strong motivation for developing automatic brake systems, highlighting the need for research and development of traffic light detection systems. Therefore, we highlight the need for research and development of traffic light detection systems that are tested in outdoor environments, consider real-world implementation challenges, and provide accurate timing information for decision-making processes, additionally to the exploration of decision-making algorithms that go beyond emergency braking actions.

## 3 Proposed methods

**Fig 1:**
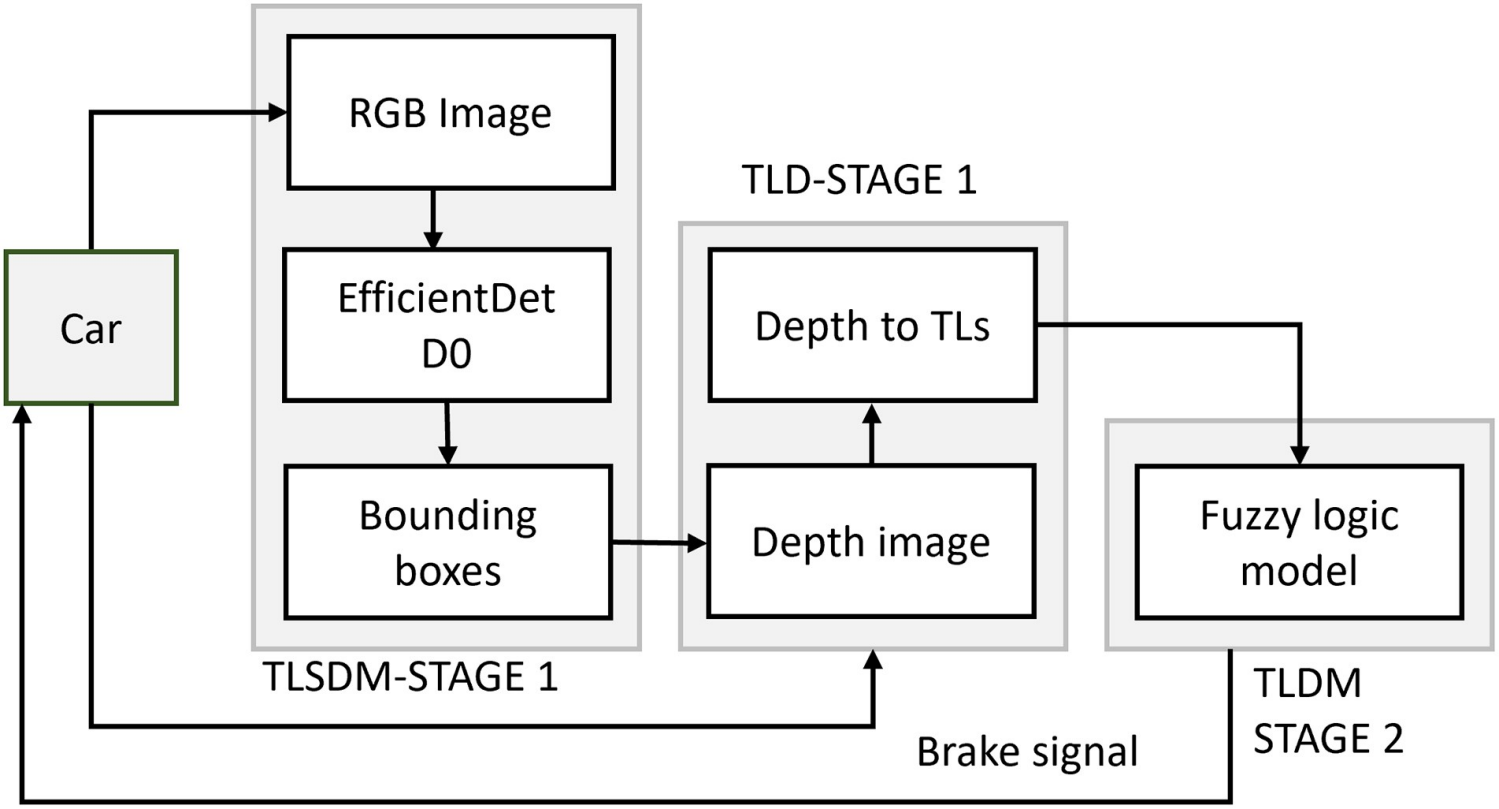
Block diagram for the proposed ABDAST. TLs: Traffic Lights; TLSDM: Traffic Light State Detection Model; TLD: Traffic Light Distance; TLDM: Traffic Light Detection Module.

A three-stage implementation of the ABDAST is proposed and implemented in a low-performance, low-power consumption embedded system. The system is a framework that uses computer vision, depth estimation, and fuzzy logic to detect traffic lights and determine the appropriate braking force for the vehicle based on the status and distance of the traffic lights in the scene (Fig 1). Each of the three stages has been converted into Robotic Operating System (ROS) nodes to enable communication between them, allowing data exchange and applying the control directive. In addition, an on-road experiment (described in Sec. 5) was performed to validate the results in a realistic environment; the test was conducted on a private road within the institution where the research was performed. The results and the automatic braking signal were measured, but no automatic braking was performed during the test, avoiding the need for specific permits related to active safety interventions.

### 3.1 Traffic light state detection model

The first stage, Traffic Light State Detection Model (TLSDM), consists of the spatial detection and state recognition of traffic lights. Traffic light detection is performed with the EfficientDet D0 detector because it is one of the lightest detectors in the EfficientDet family with only 3.9 M parameters [34]. This single-stage object detector comprises a weighted feature pyramid network and a scaling method to improve their effectiveness independently of the resolution and depth of the feature network. These models try to deal with large model sizes and expensive computational costs. Therefore, EfficientDet D0 could be an option for low-cost and low-performance embedded devices to maintain a fast response time. For this implementation, the core components, such as the EfficientNet B0 backbone architecture (Table 1), BiFPN, and the compound scaling method, were maintained the same as described in [35]. However, we have tuned the hyperparameters to achieve high performance for detecting and classifying traffic light states. Thus, the TLSDM stage works as follows:

1. RGB image information is received from the camera.
2. The EfficientDet D0 model processes each image.
3. The model provides the locations and states of traffic lights (green, yellow, red, or off) for all traffic lights in the image.
4. The bounding boxes and classes are sent to the next stage.

**Table 1:** EfficientNet B0 backbone. Conv: Convolutional Layer; MBC: Mobile Inverted Bottleneck.

Layer	Resolution	Channels	Layers
conv (3 × 3)	224 × 224	32	1
MBConv Block (3 × 3)	112 × 112	16	1
MBConv Block (3 × 3)	112 × 112	24	2
MBConv Block (5 × 5)	56 × 56	40	2
MBConv Block (3 × 3)	28 × 28	80	3
MBConv Block (5 × 5)	14 × 14	112	3
MBConv Block (5 × 5)	14 × 14	192	4
MBConv Block (3 × 3)	7 × 7	330	1
conv (1 × 1)/Pooling/FC	7 × 7	1,280	1

Then the Traffic Light Distance (TLD) system estimates the distance from the car to the traffic lights with the information from the ZED stereo camera to each traffic light detected in the previous stage. The maximum distance considered for the evaluation of the system was 15 m. This stereo camera was selected because it is low-cost and portable compared to other sensors, such as LIDAR and RADAR. In addition, it can provide an RGB image and depth estimation simultaneously, providing the user with an image to perform the detections and a depth map that contains the distance from the camera to each traffic light. Thus, the traffic light distance stage works as follows:

1. Reads the bounding boxes from the previous stage and depth map.
2. Locates the bounding boxes coordinates into the depth map.
3. Retrieves the estimated distances of the traffic lights.
4. Sends the estimated distances and states of the traffic lights to the next stage.

### 3.2 Traffic light decision making

**Fig 2:**
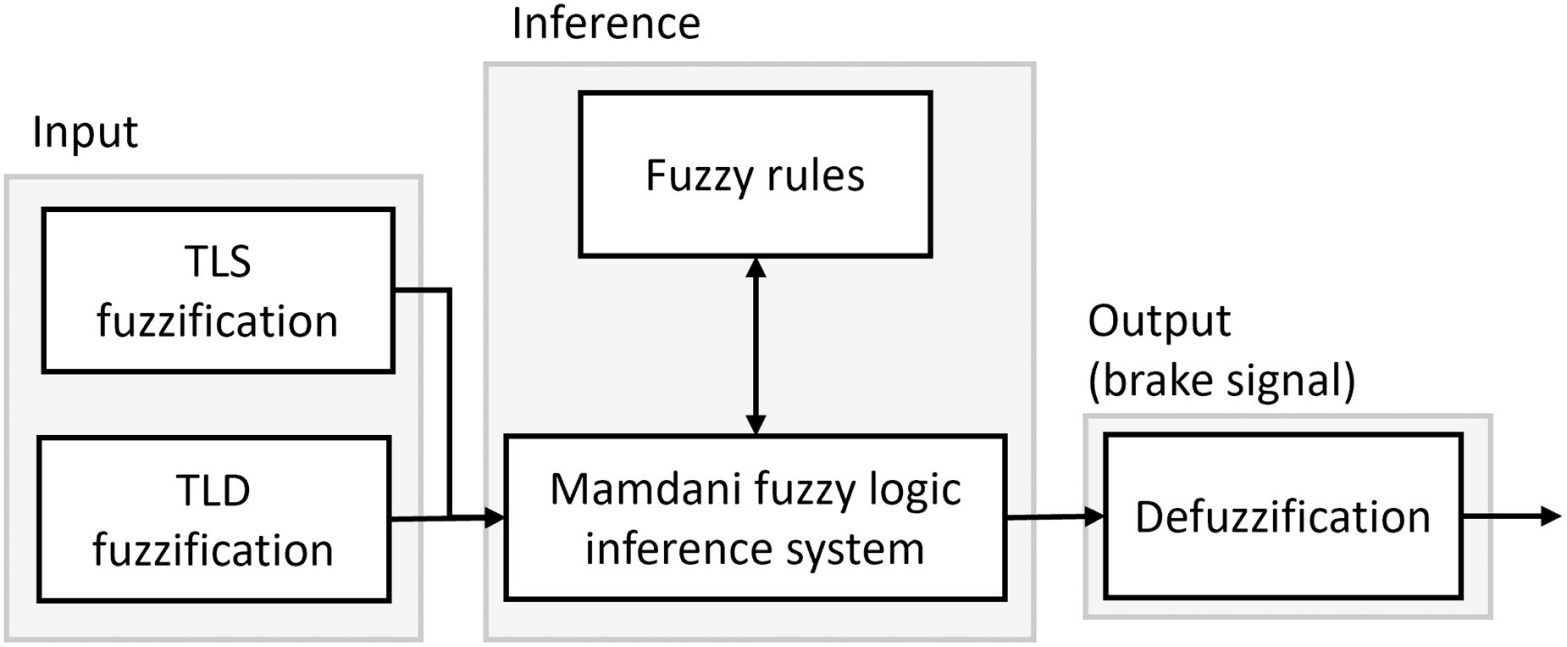
Components of the TLDM. TLS: Traffic Light State; TLD: Traffic Light Distance.

Finally, the Traffic Light Decision-Making (TLDM) applies a brake force according to two inputs: the distance in metters from 0 to 15 m, and the state of the closest traffic light (off, green, yellow, red). This last stage of the ABDAST first receives the information about the states and distance estimation from the traffic lights in the scene captured by the camera. Then the list is sorted in ascending order according with the distance from the vehicle to the traffic lights. Then, the information of the closest traffic light feed the fuzzy model to provide a brake signal that determines the degree of brake that must be applied in the current situation. The TLDM comprises three essential components: inputs, the inference model, and the output (represented as the brake signal); This system architecture is illustrated in detail in Fig 2.

**Fig 3:**
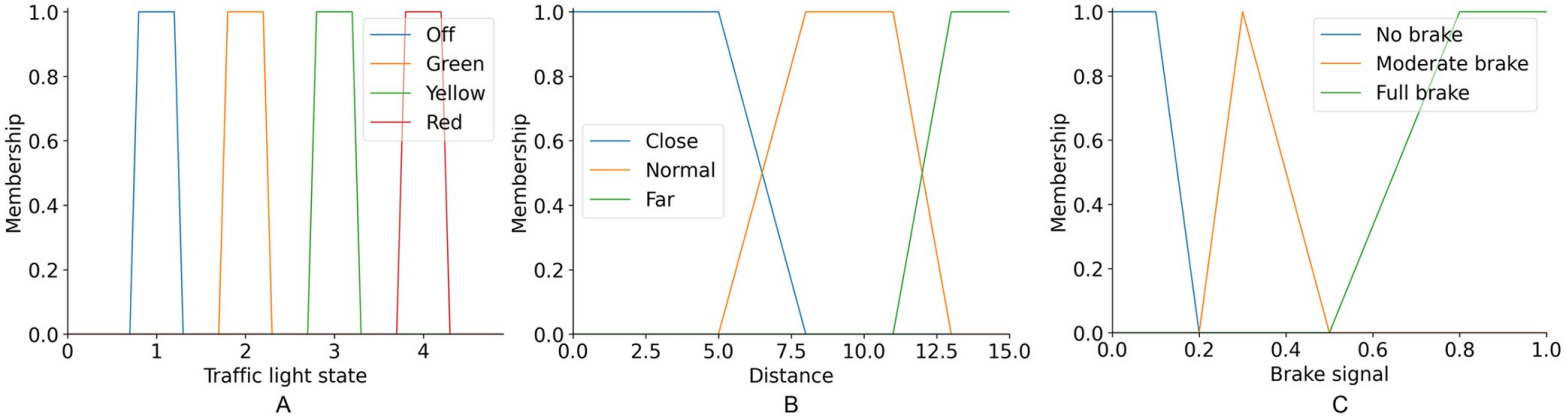
Fuzzification. Mamdami representation of the inputs/outputs of the fuzzy model.

A Mamdani fuzzy inference system (FIS) was used to implement the brake system in the skfuzzy library [36]. The implementation of the TLDM involves the design of the components shown in Fig 2, consisting of an input, a Mamdani inference system, and an output with a centroid method to provide the brake signal. The system has two inputs: the traffic light state and the distance to the closest traffic light provided by the detector. The traffic light state can contain four values represented by four fuzzy sets with trapezoidal membership functions. In contrast, the traffic light distance has continuous values ranging from 0 to 15 m, and the design includes three fuzzy sets (close, medium, and far), each represented using a trapezoidal member function with boundaries defined based on the distances used for the final experiment and empirical proofs. Finally, the brake signal (output) was modeled using three fuzzy sets named No Brake, Moderate Brake, and Full Brake. This fuzzy set contains two trapezoidal elements and a triangular membership function with values between 0 and 1, where 1 represents the maximum brake force. The linguistic variables and their definitions in fuzzy sets are shown in Table 2 and its plot in Fig 3.

**Table 2:** Linguistic variables and its definitions in terms of fuzzy sets.

Variable	Value	Function	Parameters
TLS	Off	trapezoidal	[0.75, 0.75, 1.25, 1.25]
	Green	trapezoidal	[1.75, 1.75, 2.25, 2.25]
	Yellow	trapezoidal	[2.75, 2.75, 3.25, 3.25]
	Red	trapezoidal	[3.75, 3.75, 4.25, 4.25]
TLD	Close	trapezoidal	[0, 0, 5, 8]
	Normal	trapezoidal	[5, 8, 11, 13]
	Far	trapezoidal	[11, 13, 15, 15]
BF	No brake	trapezoidal	[0, 0, 0.1, 0.2]
	Moderate brake	triangular	[0.2, 0.3, 0.5]
	Full brake	trapezoidal	[0.5, 0.8, 1, 1]

TLS: Traffic Light State; TLD: Traffic Light Distance, and BF: Brake Force

The fuzzy rules were modeled to define the behavior of the brake signal for the system using the rules presented in Table 3). Basically, when the traffic light state is “green”, or its distance is “far”, the brake signal is “No brake”; otherwise, when it is “normal”, the response is “Moderate brake”, and when it is “close”, the brake signal is “Full brake”. In this manner, the values of the applied brake force will vary according to the traffic light distance.

**Table 3:** Fuzzy rules of the traffic light decision-making system.

Rules	TLS	TLD	Brake Signal
Rule 1	Green	Close	No Brake
Rule 2	Green	Normal	
Rule 3	Green	Far	
Rule 4	Off	Far	
Rule 5	Red	Far	
Rule 6	Yellow	Far	
Rule 7	Off	Normal	Moderate Brake
Rule 8	Red	Normal	
Rule 9	Yellow	Normal	
Rule 10	Off	Close	Full Brake
Rule 11	Red	Close	
Rule 12	Yellow	Close	

TLS: Traffic Light State; TLD: Traffic Light Distance.

**Fig 4:**
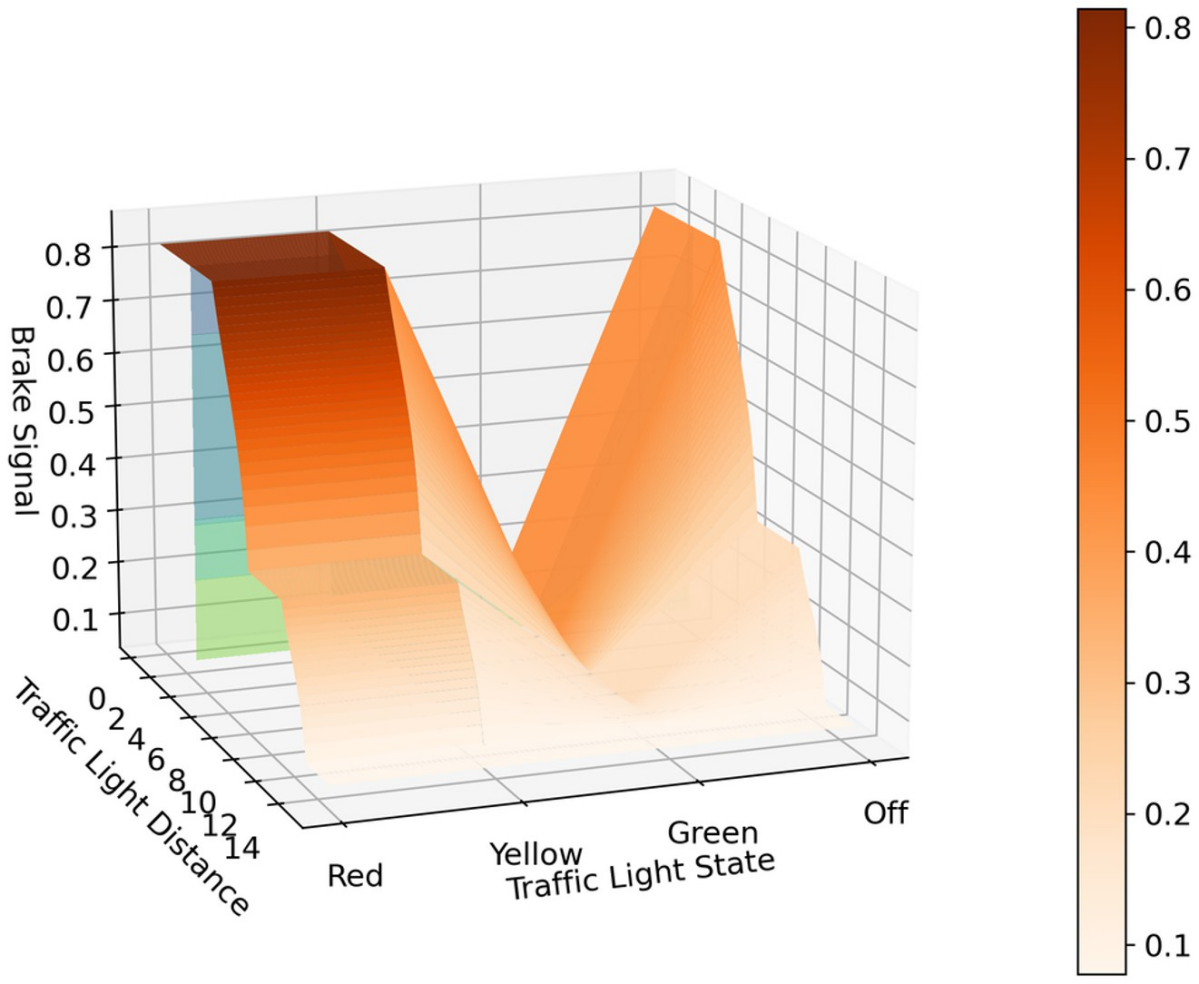
Representation of the brake signal values at different traffic light states and distances.

Fuzzy inference systems are often used to determine the brake profile of vehicles such as trains because of the smooth braking that can be achieved with them. Thus, a partition grid with possible values of the inputs fed the model, and the response for each input was obtained to know the brake profile of the proposed system. As seen in Fig 4, the response of the brake signal increases as the distance from the traffic light decreases, reaching its maximum when the traffic light is below 2 meters. On the other hand, the brake signal decreases to almost 0 when the traffic light state is green, regardless of the distance to the traffic light. The proposed fuzzy system can send a smooth braking signal along distances with higher intensities when the traffic light distance transitions from one to another membership function. This transition can be softened if more membership functions are added to the linguistic variables but at the cost of increasing the complexity of the model.

## 4 Experiments and results

### 4.1 Traffic light detection

The first experiment consists of evaluating EfficientDet D0 to detect and identify the state of traffic lights in a simplified version of the BSTLD. The BSTLD was selected for training the models based on their completeness and consistency. Published by Bosch in 2017, this public dataset is specifically designed to support the development of robust traffic light detections. It contains 13,427 annotated JPEG format images with boxes around each traffic light, containing 24,000 annotations available in JSON format. The BSTLD presented complete annotations in the dataset and provided accurate and consistent annotations for each traffic light. The annotations were taken over 17 different cities in the day-time, with varying classes including mainly green, yellow, red, and off states [4]. An exploratory data analysis was performed to analyze the characteristics of each partition, the minimum width, average width, median width, maximum width, minimum height, average height, median height, maximum height, minimum size, average size, median size, and maximum size are considered, as well as, a histogram with the new class distribution is provided (See Table 4). The 13 classes in the dataset were reduced to 4 (red, green, yellow, and off), obtaining 5093 images with 10759 annotated traffic lights for training and 834 images with 1346 annotated traffic lights for testing, covering several real scenarios such as busy streets, multilane roads, dense and not dense traffic, strong changes in illumination, light rain, among others [4]. Data augmentation techniques are applied to increase the model generalization regarding brightness, contrast, scale, and horizontal flip.

**Table 4:** Width, height, and area of the TLs from the BSTLD for each partition set. Based on the results obtained in the analysis, it can be seen that most of TLs in the BSTLD are small based on COCO metrics (area of object less than 1024 pixels).

	Width				Height				Area			
Sets	Min	Avg	Median	Max	Min	Avg	Median	Max	Min	Avg	Median	Max
Train	1.12	11.17	8.55	98	0.24	24.22	18.89	207	0.27	400.50	158.80	20286
Test	1.875	9.43	8.5	48.375	6.25	26.76	24.5	104.5	11.71	313.57	212.43	4734
Additional	2.67	10.49	8.56	50.55	3.38	23.06	18.68	113.95	12.19	323.84	164.51	5760.81

**Fig 5:**
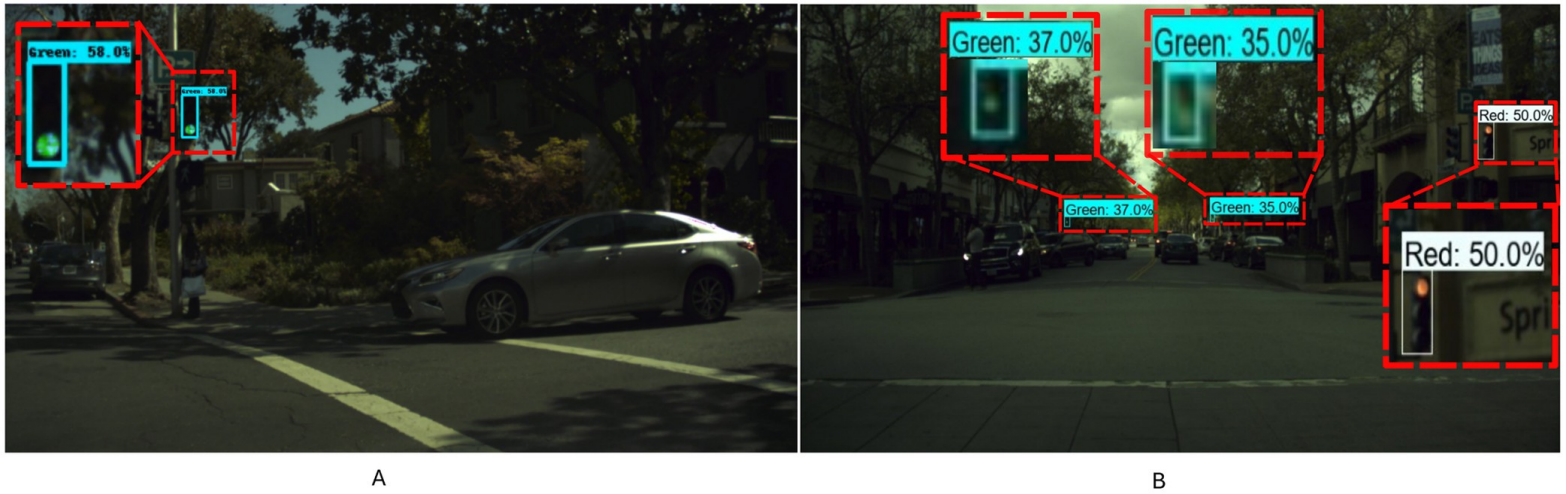
Detection examples with the EfficientDet D0 model on the BSTLD test set. (A) is a traffic light in the green state at a medium distance, and (B) two green traffic lights at far distances and a red traffic light at a medium distance.

The training was carried out with a batch size of 60 images per iteration, 12,000 training epochs, a bounding box aspect ratio of 0.33, and an exponential decay learning rate. Five data augmentation techniques were applied to avoid overfitting and improve generalization capabilities: random brightness, random contrast, random horizontal flip, random crop, and random scale. An experiment was carried out to evaluate whether the initial learning rate when training can affect the results. Three models were trained and evaluated with the test set of the BSTLD with learning rates of 0.055, 0.065, and 0.075, and some detection examples of the test set are depicted in Fig 5,. The results show that the model with a training rate of 0.055 obtained the highest mAP compared to the others with values of 0.065 and 0.075, as shown in (Table 5). Thus, this model will be used in future experiments in a real-world environment.

**Table 5:** Comparison of the mAP of the trained models for detecting traffic lights and their states. The evaluation was performed using the BSTLD [4].

	Learning rate		
Model	0.055	0.065	0.075
mAP@0.50IoU	0.307182	0.263284	0.232785
mAP@0.75IoU	0.006123	0.005365	0.009093

### 4.2 Traffic light distance estimation

**Fig 6:**
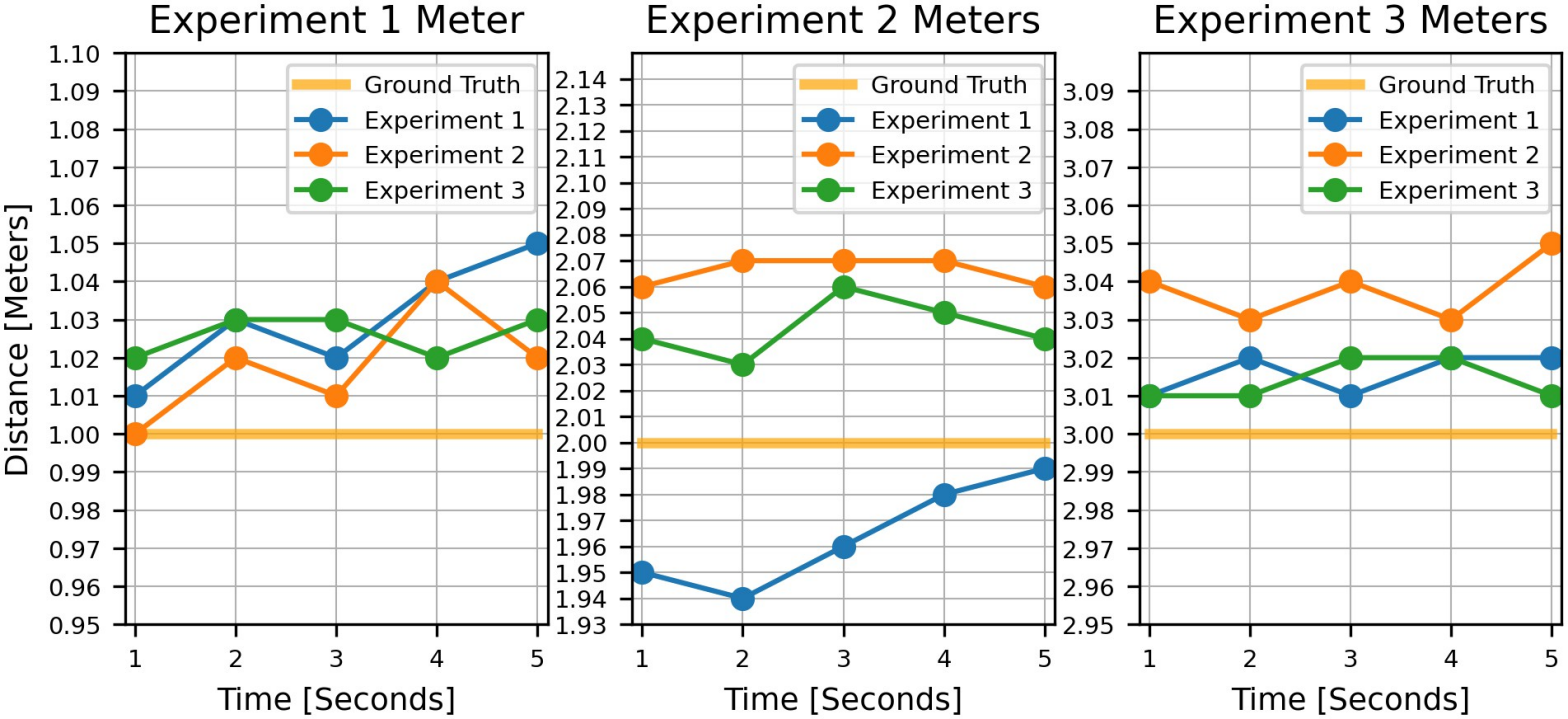
Depth estimation test using the ZED stereo camera at three different distances. Experiments 1, 2, and 3 reported a RMSE of 0.038, 0.048, and 0.029, respectively. The average RMSE of the experiments is 0.038.

The traffic light detection system returns a bounding box defined by {x, y, w, h}, where *x* and *y* are its coordinates, *w* is the width, and *h* is the height. In principle, this represents a region in the image in which the traffic light is located, but in practice, the bounding box can contain regions of other objects, especially in its periphery. Additionally, the generated depth map by the ZED stereo camera can contain noise produced by occlusions or light reflections. For these reasons, when only a pixel or a small bounding box region is picked as a sample to determine the distance to an object, the measurements often will contain a high error. Thus, an experiment determined which statistic value would measure the overall distance from the depth camera to the ROI with the least error. First, the Zed Stereo camera was calibrated using the *ZED SDK of Stereolabs ©* in a *Dell Precision m4800* computer. This calibration step uses a calibration grid and a stereo camera. The calibration kit was manipulated to face the grid, and then the software compared and connected the ROIs to the grid at different distances. To verify the calibration of the stereo camera, the depth map function from the *ZED SDK* software was executed using the *Dell Precision m4800*, which provided a real-time depth estimation. The depth map was tested in a closed environment, from the camera to an object at three different distances from 1 to 3 meters. The experiment was performed three times, and the results can be visualized in Fig 6.

**Fig 7:**
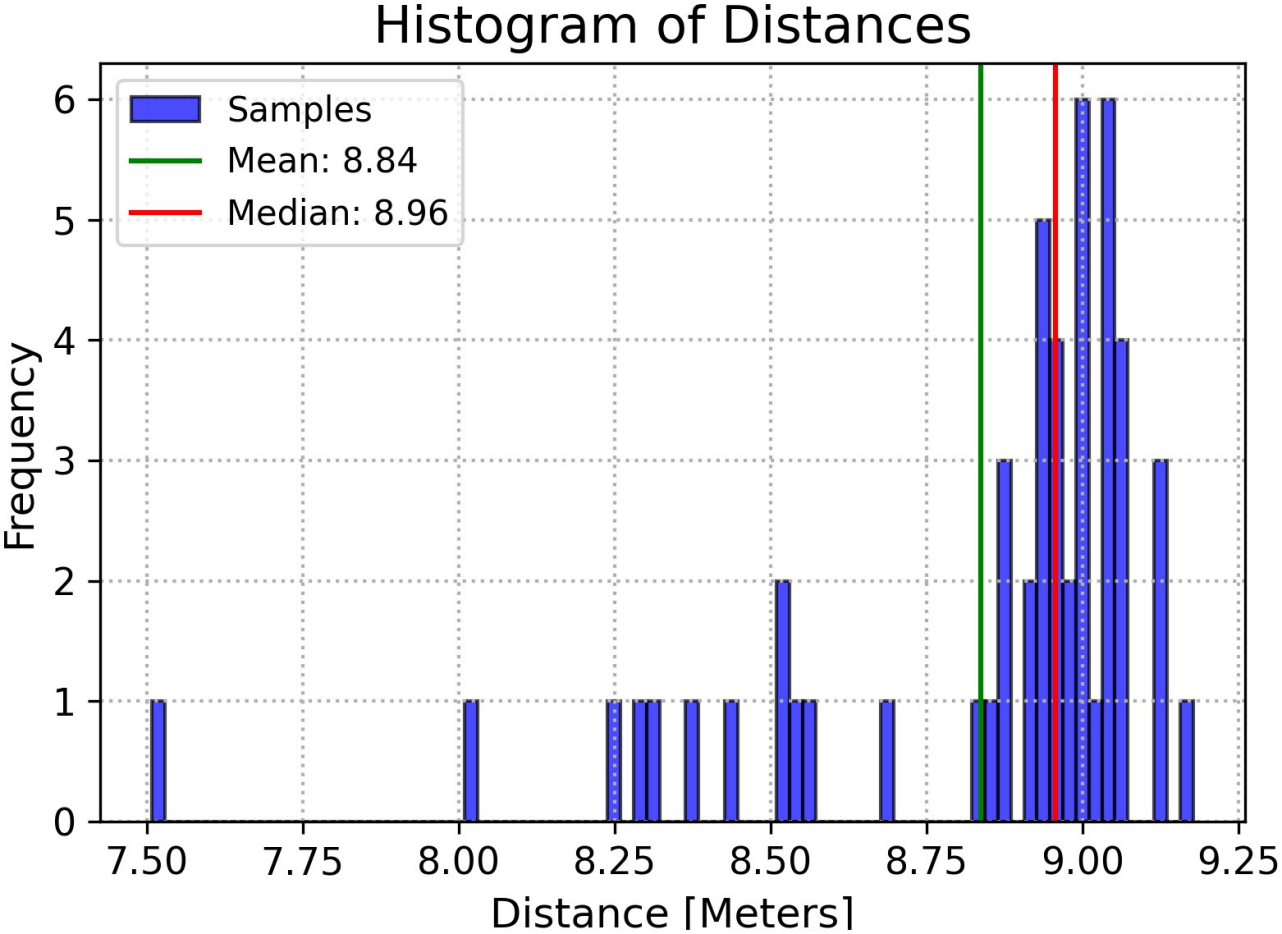
Histogram of distances from a traffic light located at 9 meters. The mean and median values are 8.84 and 8.97, respectively.

The experiment was extended to include traffic lights positioned beyond a 5-meter distance. Given the varied distance predictions, we calculated and compared the mean and median values of the pixels within the traffic light’s boundary box. For instance, when evaluating a traffic light located 9 meters away, the median yielded an average estimation of 8.97 with a mean of 8.84. This resulted in a 0.03 m error for the median and a 0.16 m error for the mean. The experimental findings suggested that the median value of all pixel values provides a more accurate estimate of the target value compared to the mean. This observation is exemplified in Fig 7.

## 5 On-road experiment

**Fig 8:**
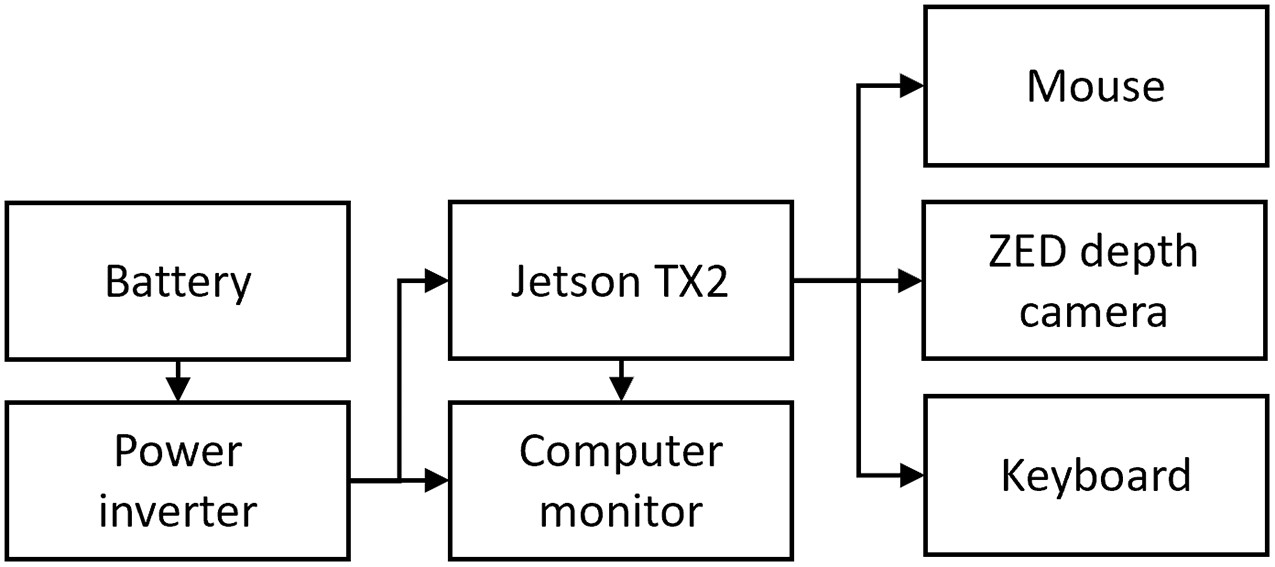
Setup of the electric components that compose the ABDAST.

**Fig 9:**
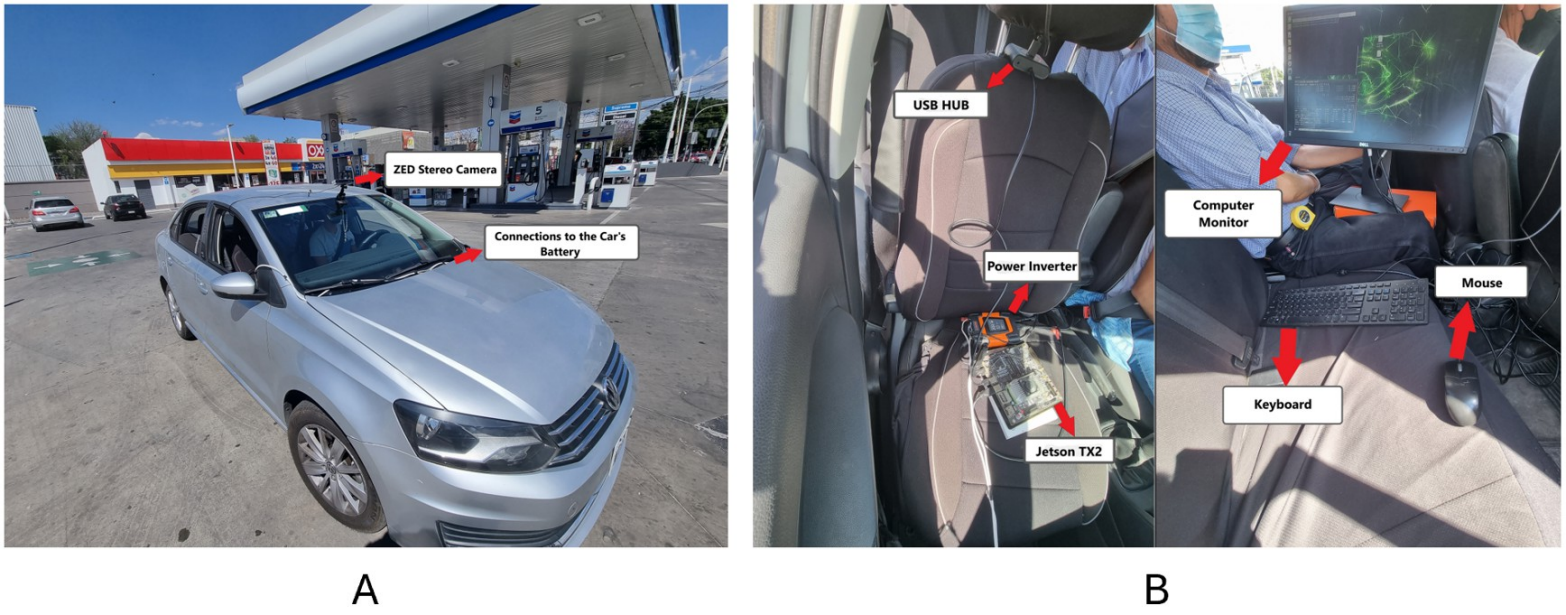
Experimental setup for the on-road test. (A) external components, and (B) internal components.

A practical on-road evaluation of the ABDAST system was conducted in a real-world setting in Mexico to assess its performance in authentic environmental conditions. The test was conducted on a private road within the institution where the research was performed (Queretaro, Mexico); for this reason, No permits were required for the described study. Distinct markers were strategically positioned on the road at intervals of 5, 7, 9, 11, 13, and 15 meters from a traffic light, enabling the systematic variation of this parameter for result evaluation. The experiments took place in a controlled environment, ensuring safety and focusing on testing the overall system’s RT. Additionally, each component of the system underwent individual testing. The road test was executed on a secure roadway, approaching vertical traffic lights at 10 km/h. For a comprehensive overview of the hardware setup please refer to Fig 8. The ABDAST software was integrated into an NVIDIA Jetson TX2 board (NVIDIA, California, United States); it has 256-core NVIDIA Pascal GPU architecture with 256 NVIDIA CUDA cores, a Dual-core NVIDIA Denver 2 + Quad-core ARM Cortex-A57 processor, 8GB LPDDR4 RAM, 32 GB eMMC 5.1 hard disc and two power modes: 7 and 15 W. The software used in the Jetson TX2 include Tensorflow 2.3.1, Bazel 3.1.0, Tensorflow Addons 0.30.0, Jetpack 4.5.1 (L4T.R32.5.1), CUDA 10.2 and cuDNN 8.0; All running in Ubuntu 18.04 LTS with python 3.6.9 and ROS2 Eloquent. The image processing takes place on the board in MAXN power mode; the processing nodes, including the depth estimation and decision-making nodes, operated on CPUs 1 to 4, while the Fuzzy inference model ran on CPUs 5 to 6. Concurrently, the depth from the ZED camera and the object detection inference node use the GPU of the NVIDIA Jetson. In contrast, the ZED stereo camera was cond with default parameters (contrast, brightness, gamma, sharpness, hue, and gamma) for RGB image acquisition. The configuration for depth map acquisition was set to “Performance quality” and “Standard sensing mode,” with a depth confidence of 50. The RGB and depth maps were obtained with an image size of 640×640 pixels. The finalized configuration of the ABDAST system, mounted on the car, is illustrated in both the external and internal electronic components (see Fig 9). The developed ABDAST components were integrated into a vehicle for comprehensive testing purposes.

The ABDAST system was subject to a comprehensive performance evaluation. The mAP was employed across various IoU thresholds, assessing the TLSDM. Additionally, the TLD was appraised through the Root Mean Squared Error (RMSE) between the predicted and actual distances of the traffic lights. The TLDM component underwent evaluation using the RMSE between predicted and expected brake signals. Furthermore, the overall system performance was gauged by summing the response times RTs at each stage of the ABDAST.

Notably, the TLSDM exhibited a mAP@0.50IoU surpassing 0.96 for distances below 13 meters. Beyond this threshold, during the on-road experiment (see Table 6), the mAP@0.50IoU decreased to nearly 0.90. This outcome signifies the TLSDM’s consistent ability to detect green and red traffic light states within the 5 to 15 m range. Notably, this stage is the most computationally demanding among all ABDAST components, with an inference time of approximately 0.20 seconds using the NVIDIA Jetson TX2.

**Table 6:** The mAP of the red and green traffic light states reported for small, medium, large, and all object sizes at six different landmark positions.

Experiments	mAP@0.50IoU	mAP@0.75IoU
5 Meters	1.0000	0.6942
7 Meters	1.0000	0.5126
9 Meters	0.9703	0.1381
11 Meters	0.9677	0.0639
13 Meters	1.0000	0.0560
15 Meters	0.8950	0.0515

**Fig 10:**
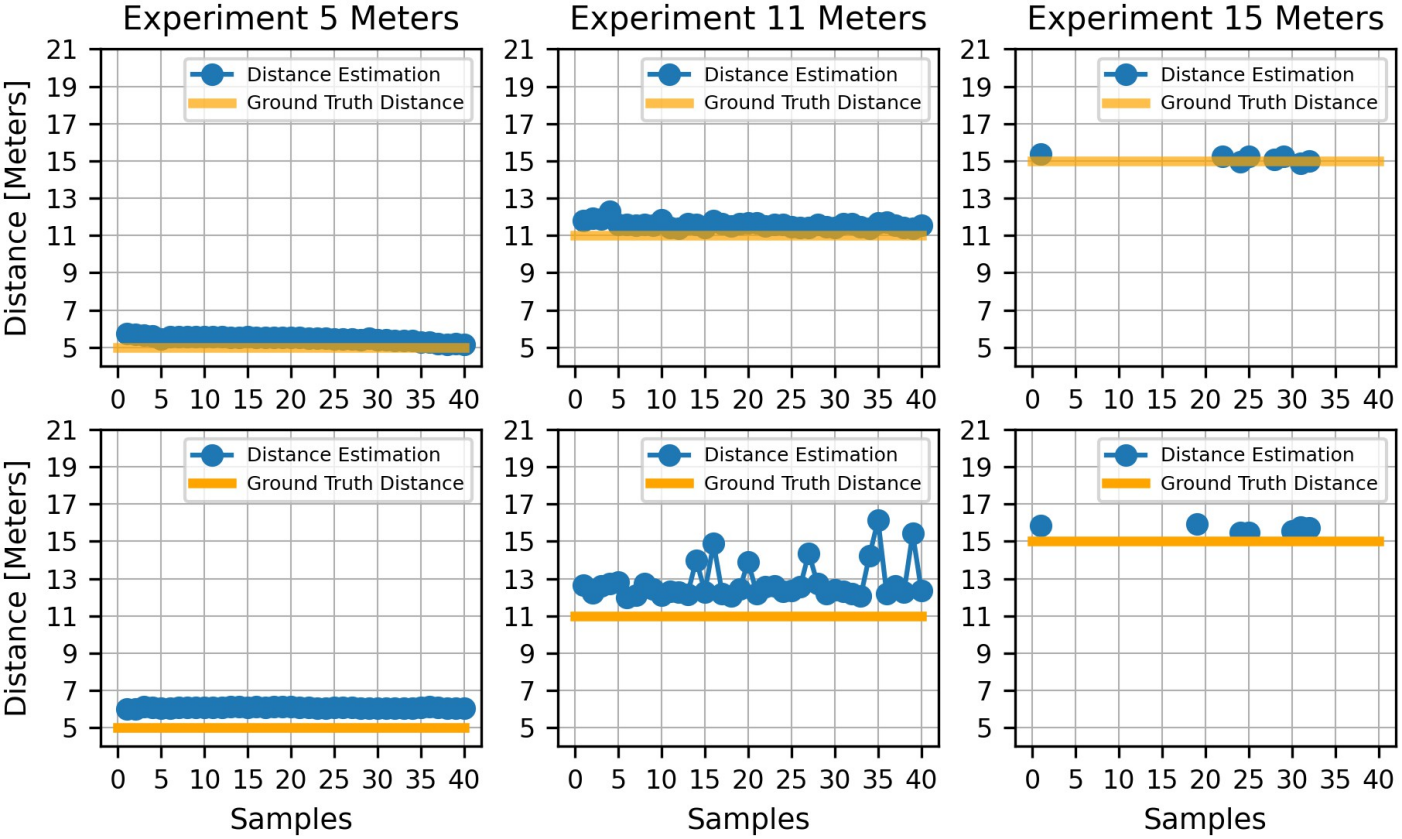
Distance estimation provided by the ABDAST versus distance ground truth for each landmark. The first and second rows correspond to the values obtained during the green and red traffic light states experiments.

For the TLD, the RMSE of the distance between the car and the traffic light was calculated using the landmarks. The results (Fig 10) indicate that it is possible to locate and estimate the depth to it with an error of 1 m (Table 7).

**Table 7:** RMSE from the on-road experiment of the distance and brake signal ground truth, and distance and brake signal estimated values.

TL State	GT Distance (m)	Ground Truth Brake Signal	Distance RMSE (m)	Brake Signal RMSE
Green	5	0.08	0.4896	0.0012
Red		0.81	1.0767	0.1309
Green	7	0.08	0.7166	0.0007
Red		0.57	1.0924	0.2345
Green	9	0.08	0.6888	0.0022
Red		0.33	0.8770	0.0033
Green	11	0.08	0.6246	0.0039
Red		0.33	2.0190	0.1684
Green	13	0.08	2.2238	0.0029
Red		0.08	0.5526	0.0691
Green	15	0.08	0.2096	0.0022
Red		0.08	0.7014	0.0022

TL: Traffic Light; GT: Ground Truth.

**Fig 11:**
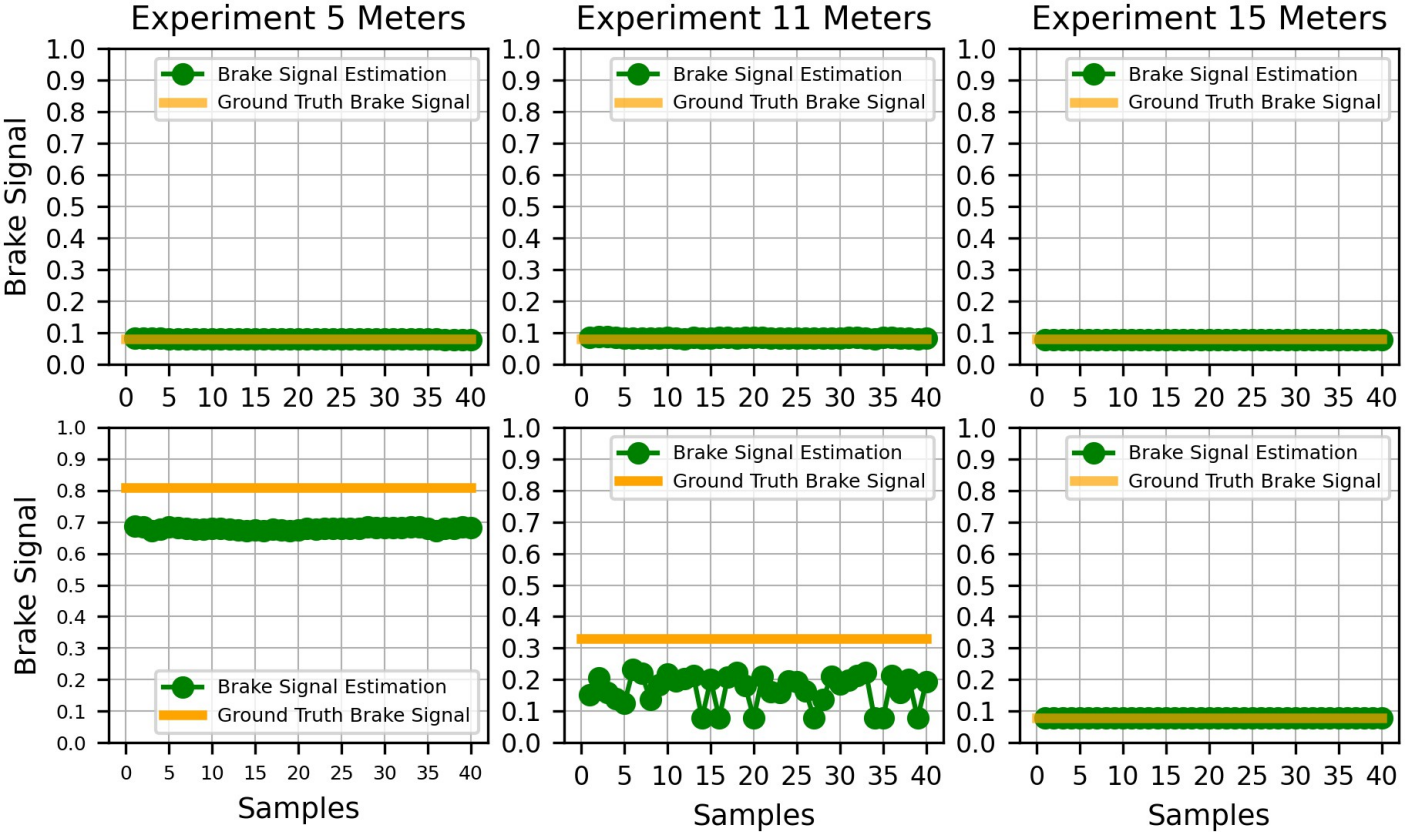
Brake estimation provided by ABDAST vs. brake ground truth for each landmark. The first and second rows correspond to the values obtained during the green and red traffic-light states experiments.

Finally, to assess the effectiveness of TLDM, the fuzzy logic model values were computed for each landmark. These values were then compared with the output from TLDM in the experimental setting, and the results are illustrated in Fig 11. The RMSE for both brake signal and traffic light distance estimations are presented in Table 7. The on-road experiments revealed that, despite unattainable estimates and considerable variations in traffic light distance, the designed brake profile demonstrated commendable performance.

## 6 Conclusions and future work

We have introduced a novel solution for an Automatic Brake System Based on Traffic Lights using EfficientDet D0 and fuzzy logic in this research. The system deployed on an NVIDIA Jetson TX2 operates at 0.23 seconds or 4 Hz, exhibiting effective and accurate performance in a real-world scenario in Mexico. In this section, we will highlight the most pertinent findings of our study and compare these results with existing solutions. The selected model for TLSDM presented a value of 0.3072 mAP @0.50IoU using the BSTLD, while Behrendt et al. [4] reported a mAP@0.50IoU of 0.40 using a two-stage traffic light detector, and Lee et al. [19] provided a mAP@0.50IoU of 0.57 for traffic light detection. However, the mAP reported by [4, 19] is based on the detection of the traffic light of the object rather than the detection of the traffic light state as in our solution, and the results cannot be directly compared because different databases were used for training and testing. The proposed ADAS achieved a 96% mAP@50 for traffic light detection and state recognition and an average RMSE of 1 m for distance estimation in our on-road experiment. However, the system had difficulties in calculating the distance of traffic lights at distances beyond 13 meters. On the other hand, the TLDM module exhibited an average RMSE of 0.05 for the brake signal, comparing the output of the proposed Mamdani fuzzy inference system with the expected output. The reaction time of 0.23 s indicates that ADAS is feasible for an embedded system with low resources. Thus, the results suggest that EfficientDet D0 and deep learning models embedded in a Jetson TX2 have potential in this application.

On the other hand, Wael et al. estimated the distance to the traffic lights with the ZED Stereo camera, obtaining an error of 11.49 m for 24 m and 2.4 m for 13 m. In contrast with the proposed configuration, our method is more precise in close distances, but for distances greater than 13 meters, some of the depth maps generated for the traffic lights were not able to provide a distance estimation, mainly for the lights and shadows present in the environment, as shown in (Fig 10). On the other hand, Cabrera et al., in their study, presented a method for estimating traffic light distance using a monocular camera. Their approach relies on assuming a constant height for the traffic light in order to estimate its distance.

The proposed design performs well during the on-road experiment at up to 10 km/h velocities. The hardware components used during this research were low-budget and low-performance compared to those in the related work. For this reason, experiments at velocities higher than 10 km/h were not considered. Nevertheless, the system performance at velocities higher than 10 km/h can be inferred; the system currently has a response time of 0.23 s, from which the TLSDM takes 0.20 s. At a speed of 10 km/h (2.5 m/s), the system updates the required braking degree every 0.625 meters. For higher speeds, such as 20 km/h, decisions need to be performed every 1.25 meters. Therefore, the system is limited by its first stage: the TLSDM. Thus, several solutions can be implemented to address these challenges in future work to ensure safer and more reliable operation in practical autonomous driving applications: Convert the EfficientDet D0 model to TensorRT, optimizing and accelerating the deployment of trained neural networks on NVIDIA GPUs. This would increase the response time twice, as seen in the literature. Also, Upgrade from the NVIDIA Jetson TX2 to a more powerful device used in the automotive industry, such as the NVIDIA DRIVE Orin. This upgrade would dramatically decrease the inference time of the model, thereby reducing the overall response time of the system.
